# Phenology, Canopy Aging and Seasonal Carbon Balance as Related to Delayed Winter Pruning of *Vitis vinifera* L. cv. Sangiovese Grapevines

**DOI:** 10.3389/fpls.2016.00659

**Published:** 2016-05-13

**Authors:** Matteo Gatti, Facundo J. Pirez, Giorgio Chiari, Sergio Tombesi, Alberto Palliotti, Maria C. Merli, Stefano Poni

**Affiliations:** ^1^Dipartimento di Scienze delle Produzioni Vegetali Sostenibili, Università Cattolica del Sacro CuorePiacenza, Italy; ^2^Dipartimento di Scienze Agrarie, Alimentari e Ambientali, Università di PerugiaPerugia, Italy

**Keywords:** *Vitis vinifera* L., gas exchange, source-sink balance, leaf age, photosynthesis, bud development

## Abstract

Manipulating or shifting annual grapevine growing cycle to offset limitations imposed by global warming is a must today, and delayed winter pruning is a tool to achieve it. However, no information is available about its physiological background, especially in relation to modifications in canopy phenology, demography and seasonal carbon budget. Mechanistic hypothesis underlying this work was that very late winter pruning (LWP) can achieve significant postponement of phenological stages so that ripening might occur in a cooler period and, concurrently, ripening potential can be improved due to higher efficiency and prolonged longevity of the canopy. Variability in the dynamics of the annual cycle was created in mature potted cv. Sangiovese grapevines subjected to either standard winter pruning (SWP) or late and very late winter pruning (LWP, VLWP) performed when apical shoots on the unpruned canes were at the stage of 2 and 7 unfolded leaves. Vegetative growth, phenology and canopy net CO_2_ exchange (NCER) were followed throughout the season. Despite LWP and VLWP induced a bud-burst delay of 17 and 31 days vs. SWP, the delay was fully offset at harvest for LWP and was reduced to 6 days in VLWP. LWP showed notably higher canopy efficiency as shorter time needed to reach maximum NCER/leaf area (22 days vs. 34 in SWP), highest maximum NCER/leaf area (+37% as compared to SWP) and higher NCER/leaf area rates from veraison to end of season. As a result, seasonal cumulated carbon in LWP was 17% higher than SWP. A negative functional relationship was also established between amount of leaf area removed at winter pruning and yield per vine and berry number per cluster. Although retarded winter pruning was not able to postpone late-season phenological stages under the warm conditions of this study, it showed a remarkable potential to limit yield while improving grape quality, thereby fostering the hypothesis that it could be used to replace time-consuming and costly cluster thinning. This preliminary study indicates that proper winter pruning date should be timed so as not to exceed the stage of two unfolded leaves.

## Introduction

The relationship between leaf age and function in grapevine has been extensively studied in the past (Kriedemann, 1968; Poni et al., 1994; Patakas et al., 1997). A common feature of these studies, which were carried out under varying growing conditions and for different cultivars, is a rapid increase in leaf net photosynthesis (P_n_) from unfolding to 35–40 days of age that attends a rapid surge in chlorophyll and nitrogen leaf concentration. Once the peak is reached, P_n_ rates thereafter usually start to decline at a rate depending upon environmental conditions, leaf exposure and source-to-sink balance (Chaumont et al., 1994). Working on Sangiovese vines grown without irrigation in the warm Po Valley, Poni et al. (1994) found that at 4 months of age leaf P_n_ rates were halved compared to maximum rates reached at about 45 days; conversely, Schultz et al. (1996) found that maximum P_n_ rates in Riesling vines grown in the cooler Rhine Valley stayed almost constant for approximately 3 months after reaching a peak at ∼30 days of age.

Benefits derived from clarification of the above relationships are apparent since knowing how the P_n_ vs. leaf age relationships works makes it easier to decide the right time and severity of summer pruning. Shoot trimming is indeed the benefit having the most profound effects on seasonal canopy demography and function. In fact, when shoots are trimmed, the canopy abruptly “ages” since the youngest leaves are removed but lateral regrowth triggered by shoot cuts rejuvenates the canopy and, eventually, the newly formed lateral leaves can become an important source for ripening (Poni and Giachino, 2000).

From a methodological standpoint, seasonal canopy demography and function are quite difficult processes to be studied since they depend upon changes in several factors inherent to the “population” of leaves composing the canopy, including age, light exposure, health status, shedding, and so forth. Inferring or extrapolating such complex interactions from single leaf-based assessment can lead to very rough approximations (Tarara and Peña, 2015). Good tools to achieving more reliable data are either modeling (Wermelinger et al., 1991; Cola et al., 2014) or the use of whole canopy gas-exchange systems (Garcia et al., 1990; Poni et al., 2014) since they can not only integrate multiple factors intervening at the canopy level in one reading but can be set up and maintained in place throughout the season, thereby delivering the needed continuous, long-term monitoring and evaluation of the entire process.

Canopy demography and function can also be changed according to the type and timing of winter pruning. For instance, the high vine-node number retained by hedge or minimal pruning is known to alter canopy filling and demography (Sommer et al., 1995). In a trial carried out on Chardonnay (Poni et al., 2000), minimally pruned vines exhibited a 4–6-fold higher CO_2_ fixation than hand pruned (HP) vines from about 3 weeks after bud burst to bloom. Canopy NCER started to recover in HP vines concurrently with the transition to a faster shoot growth phase; although by canopy completion NCER was still 13% higher in MP.

Resorting to unusually late winter pruning (LWP) can be justified on the basis of a trend toward earlier than usual grape ripening marked by excessive and/or overly fast berry total soluble solids (TSS) accumulation and detrimentally high alcohol content in the resulting wine which has been reported in several countries (reviewed in Palliotti et al., 2014); alcohol concentration in Australian red wines has increased approximately 1% v/v per decade since the 1980s, for example (Varela et al., 2015). In many instances, and regardless of cultivar, these phenomena have also been coupled to unacceptably low total acidity (TA), high pH and atypical grape flavors shifting toward overripe and jammy attributes (Keller, 2010). This scenario has been the fillip for very active crop-management research to reduce ethanol concentration in wines (Varela et al., 2015). One promising practice among those proposed is post-veraison leaf removal in the upper two–thirds of a vertical canopy, i.e., apical to the cluster zone. Once again, the technique originates from the well-known physiological background of the leaf age-leaf function relationship in grapevine: if ripening delay is sought, the rationale is that the most functional leaves have to be removed using a strategy that is the opposite of conventional basal-leaf removal. Poni et al. (2013) showed that TSS content in cv. Sangiovese grape must and wine alcohol concentration was significantly reduced (the latter by 0.6% v/v) without any significant effect on other compositional parameters, including phenolic compounds. On the same variety, Palliotti et al. (2013) recommend mechanical leaf removal in a 50-cm window at 15–16° Brix to remove 30–35% of the leaf area. This practice can be done faster than conventional cluster-zone leaf removal and has the extra advantage, in a warm climate, that cluster zone exposure is minimally affected.

Delayed vine pruning in late winter or early spring has been studied since the second half of the last century (Antcliff et al., 1957), its primary aim being to postpone bud burst by few days and, hence, to increase the chances of escaping damaging spring frost in cool-climate growing areas (Howell and Wolpert, 1978). A more delayed spur-pruning (i.e., performed at the swollen bud phenological stage or later) is expected to modify vegetative growth and shift flowering, fruit-set and all subsequent phenological stages and impact fruit chemical composition at harvest. Friend and Trought (2007) have shown that very late pruning performed on Merlot grown in New Zealand when apical shoots on canes were about 5 cm long (i.e., October vs. the usual pruning time of July) caused lower sugar and higher organic acids content in grapes. Yet, to the best of our knowledge, no physiological assessment of how canopy demography, phenology and function are modified upon very late winter pruning (VLWP; i.e., when vegetative growth has already commenced) is currently available in literature. In lack of this basic information, calibration of the technique in regard to timing and modalities looks rather troublesome. Thus, the purposes of the present study were to: (a) determine if and how phenology and shoot growth and age are affected by LWP as compared to the standard practice; (b) assess seasonal whole-canopy net CO_2_ exchange rate in each treatment; and (c) provide estimates of seasonal carbon budget and establish preliminary correlations with yield components and final grape composition. Mechanistic hypothesis underlying this work was that VLWP can achieve significant postponement of phenological stages so that ripening might occur in a cooler period and, concurrently, ripening potential can be improved due to higher efficiency and prolonged longevity of the canopy.

## Materials and Methods

### Plant Material and Treatment Layout

The trial was conducted in 2015 at Piacenza (45°02′N, 9°43′E), Italy, on 5-year-old spur-pruned cv. Sangiovese vines (*Vitis vinifera* L) grafted on SO4 and grown outdoors in 40 L pots. Twelve vines were arranged along a single, vertically shoot-positioned, 35° NE-SW oriented row and hedgerow-trained. Each vine had a ∼1 m long cordon bearing six 2-count node spurs that was raised 90 cm from the ground with three pairs of surmounting catch wires for a canopy wall extending about 1.3 m above the main wire. The pots were filled with a mixture of sand, loam and clay (65, 20, and 15% by volume, respectively) and kept well-watered throughout the whole trial. Pots were pale-green colored to limit radiation-induced overheating and each vine was fertilized twice (i.e., 1 week before and 2 weeks after respective bud-break dates) with 4 g of Greenplant 15 (N) + 5 (P_2_O_5_) + 25 (K_2_O) + 2 (MgO) + micro (Green Has Italia, Cuneo, Italy).

**FIGURE 1:**
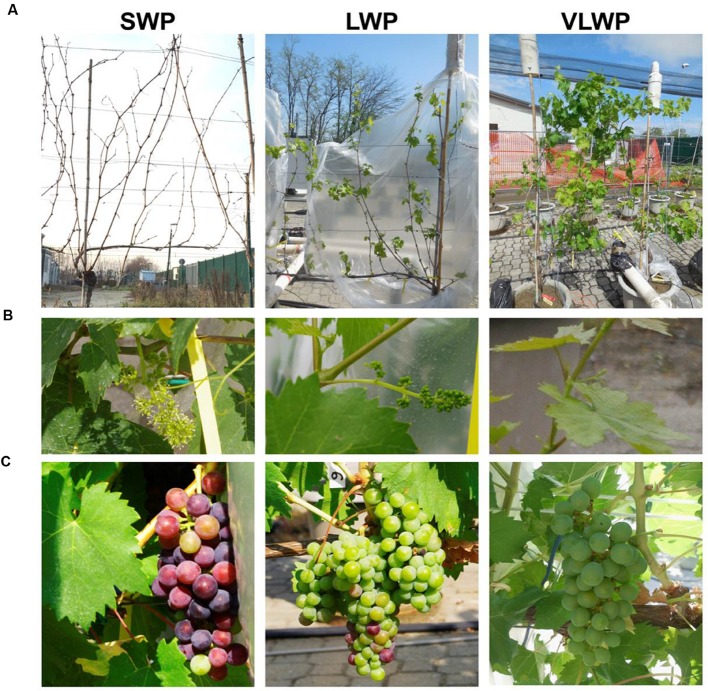
**Pictures showing vine appearance at the time winter pruning was performed (A), at BBCH-65 (B, SWP taken as reference) and at BBCH-81 (C, SWP taken as reference)**.

In winter, the vines were arranged in four blocks each of three vines and each vine randomly assigned to (a) standard winter pruning (SWP) performed on 19 February when all buds were still dormant, (b) LWP performed when, on average, the two apical shoots on the unpruned canes were at the BBCH-12 (i.e., two leaves unfolded according to Lorentz et al., 1995), and (c) VLWP performed when, on average, the two apical shoots on the unpruned canes were at the BBCH-17 stage (i.e., seven leaves unfolded corresponding to BBCH-53) (**Figure 1**). Dates of LWP and VLWP were 13 April (DOY 103) and 29 April (DOY119), respectively. Due to asynchrony in canopy development, shoots were trimmed on 1 July (DOY 182) in both SWP and LWP, whereas trimming was performed 2 weeks later (DOY 197) on VLWP. Four of the six spurs retained per vine were enclosed in chambers for continuous gas-exchange monitoring; the remaining two (i.e., those inserted at distal cordon positions) were left un-chambered in order to assure free access for detailed growth and phenology readings.

### Vegetative Growth, Phenology, Yield Components and Grape Composition

On 20 May the leaf area of 10 shoots randomly collected from additional Sangiovese vines was determined by measuring the surface of each lamina via a leaf area meter (LI-3000A, LI-COR Biosciences, Lincoln, NE, USA). Shoot length (y), ranging between 10 and 150 cm, was correlated with the corresponding shoot leaf area (x) and the resulting regression equation *y* = 0.67x^2^ + 2.76x, *R*^2^ = 0.90 was then used for daily estimates of canopy leaf area. Values were adjusted ex-post according to final actual single leaf surface determined per treatment at the end of the trial.

The main leaves inserted on nodes 3, 6, 9, 12, 15, 18, and 21 of the distal shoot of both un-chambered spurs were collected on each vine at harvest and their surface measured with the leaf area meter. At the same time mean lateral leaf size was determined on a representative sample randomly collected from laterals. Immediately after leaf fall, total nodes per cane were counted as well as the number of nodes of each lateral shoot; final leaf area per vine was then estimated on the basis of node counts and leaf blade areas.

Chambers were temporarily dismantled for harvest at BBCH 89 on 26 August (SWP and LWP) and 4 September (VLWP). Each vine was individually picked and all clusters were counted and weighed, considering the six spurs as sub-replicates. The number of seeded and seedless berries was counted, rachis length measured and total berries per cluster were computed as the sum of seeded and seedless grapes, excluding shot berries. Cluster compactness was then calculated and expressed both as total berry fresh mass and total berries/rachis plus main wing length ratios (Tello and Ibanez, 2014).

A 50-berry sample was taken from each of the six sub-samples per vine at harvest to ensure that the positions within the cluster (top, mid, bottom) and exposures (internal or external berries) were represented; these samples were then weighed and stored at -20° C for subsequent analyses. All the remaining crop was crushed, must TSSs concentration (°Brix) determined and titratable acidity (TA) measured by titration with 0.1 N NaOH to a pH 8.2 end point and expressed as g/L of tartaric acid equivalents. Tartrate was assessed on must via the colorimetric method based on silver nitrate and ammonium vanadate reactions (Lipka and Tanner, 1974). Malate was determined with a kit (Megazyme Int., Bray, Ireland) that uses L-malic dehydrogenase to catalyze the reaction between malate and NAD^+^ to oxaloacetate and NADH. The reaction products were measured spectrophotometrically by the change in absorbance at 340 nm from the reduction of NAD^+^ to NADH. Potassium (K^+^) concentration was measured in the must using an ion-selective electrode (Model 96-61, Crison, Carpi, Italy).

Anthocyanins and phenolic substances were determined after Iland (1988) using the 50-berry sample left to thaw and then homogenized at high speed (7602 *g*) with an Ultra-Turrax (Rose Scientific Ltd, Edmonton, AB, Canada) homogenizer for 1 min. Two grams of the homogenate were transferred to a pre-tarred centrifuge tube, enriched with 10 mL aqueous ethanol (50%, pH 5.0), capped and mixed periodically for 1 h before centrifugation at 959 *g* for 5 min. A portion of the extract (0.5 mL) was added to 10 mL 1 M HCl, mixed and let stand for 3 h; the absorbance values were then measured at 520 and 280 nm on a JascoV-530 UV spectrophotometer (Jasco Analytical Instruments, Easton, MD, USA). Anthocyanins and phenolic substances were expressed as mg/g of FM and mg/berry.

### Whole-Canopy Gas Exchange

Whole-canopy net CO_2_ exchange rate (NCER) measurements were taken using the multi-chamber system reported in Poni et al. (2014). In order to capture whole-season carbon balance changes in each treatment, the chambers were set up on each vine when all buds were still dormant (13 March, DOY 72) and continuously operated 24 h per day until 5 November (DOY 309) when leaf shedding had already started. Chambers were temporarily dismantled three times during the season for periods never exceeding a week in length to allow sprays, canopy management operations and manual harvest. The flow rate fed to the chambers was progressively adjusted according to the increasing leaf area enclosed in the chambers and varied from 2.8 L/s set at the beginning of growth to 15.6 L/s imposed when canopies reached their final size. Ambient (inlet) air temperature and the air temperature at each chamber’s outlet were measured by shielded 1/0.2 mm diameter PFA –Teflon insulated type-T thermocouples (Omega Eng. INC, Stamford, Connecticut); direct and diffuse radiation were measured with a BF2 sunshine sensor (Delta-T Devices Ltd., Cambridge, UK) placed horizontally on top of a support stake next to the chambers enclosing the canopies. Canopy NCER (μmol CO_2_/s) was calculated from flow rates and CO_2_ differentials after Long and Hallgren (1985).

### Data Analysis

One-way analysis of variance was carried out and, in case of significance of *F*-test, mean separation was performed by the Student–Newman–Keuls test at *P* < 0.05 and 0.01. BBCH readings were transformed into root squared values prior to analysis. Table-Curve 2D (Systat Software Inc., London, UK) was use to run polynomial regressions. Degree of variation around means is given as standard error (SE).

## Results

### Phenology and Demography

**FIGURE 2:**
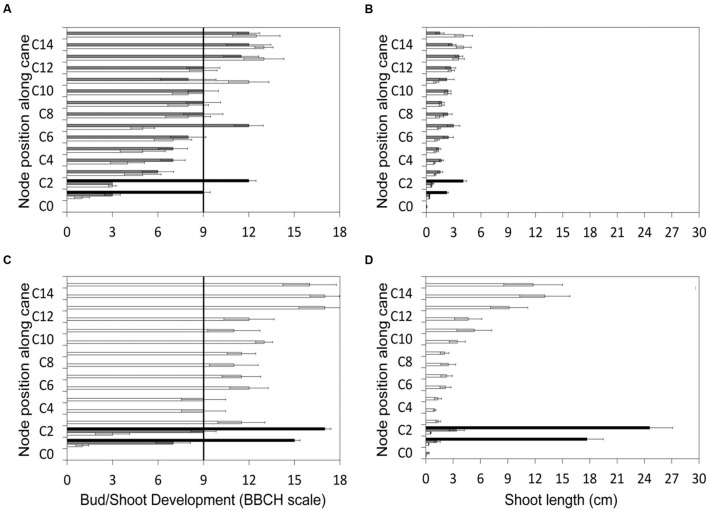
**Gradient of canopy growth along the cane recorded for the three pruning treatments before late winter pruning (LWP) (top panels) and very late winter pruning (VLWP) (bottom panels).** Growth is given both as BBCH phase **(A,C)** and shoot length **(B,D)**. C_n_ represents node position along the cane. Vertical bars on the left side of panels indicate bud-burst according to BBCH scale (Lorentz et al., 1995).

The gradient of vegetative growth along the unpruned canes of LWP indicates that prior to pruning performed on DOY 103, the two apical shoots had reached the BBCH stage of two unfolded leaves, corresponding to a shoot length of about 4 cm (**Figures 2A,B**). The two basal LWP nodes to be retained with spur pruning were at the end of the bud swelling stage (BBCH-03). The same day acrotony was also exhibited by the two-node SWP spurs, the distal one having already reached the two unfolded leaf-stage and the proximal one still lagging at bud burst. As expected, the growth gradient recorded immediately before VLWP pruning on 29 April (DOY 119) was more pronounced with the two apical shoots at 6–7 unfolded leaves and at 12–14 cm in length, also corresponding to BBCH-53 (**Figures 2C,D**). By analogy to LWP, the two basal nodes of VLWP were still at bud swelling stages when pruning was performed on DOY 119.

**FIGURE 3:**
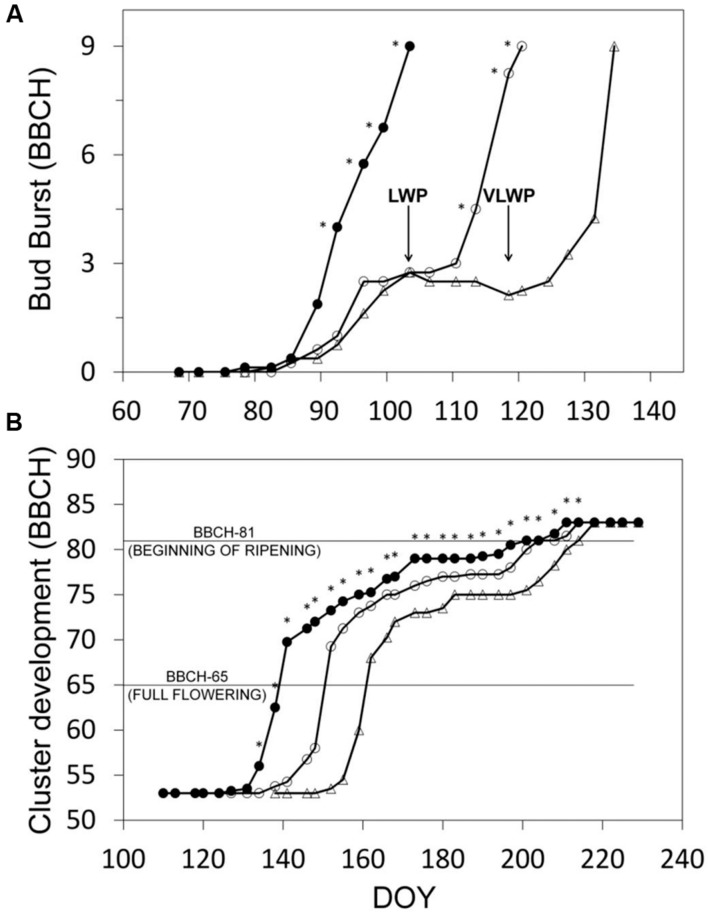
**Progression to bud-burst (BBCH-09) (A) and through the reproductive stages (B) for the three pruning treatments.** Estimates in **(B)** fall within the interval between BBCH-53 (inflorescences clearly visible) and BBCH-83 (berries developing color). In **(A)**, arrows indicate DOY of winter pruning for LWP and VLWP treatments. In **(B)**, within date, asterisk indicates mean separation at p ≤ 0.05 by SNK or *t*-test according to the number of treatments (3 or 2, respectively). No asterisk means ns.

Progression trends toward budburst showed that while bud opening in SWP was closely related to chronological time (from DOY 82, *y* = 0.442x-36.806, *R*^2^ = 0.98), apical dominance in LWP kept the two basal buds at bud-swelling stages for 2 weeks prior to pruning and it took about 10 days to reactivate development after pruning (**Figure 3A**). In VLWP, the two basal nodes remained at bud swelling for approximately a month (DOY 89–119) and post-pruning resumption of bud development occurred in approximately 10 days. This dynamic led to a bud-burst delay of 17 and 31 days for LWP and VLWP vs. SWP, respectively (**Table 1**). The tendency in both late pruning treatments was to reduce this delay over the progressing season (**Figure 3B**; **Table 1**). At full flowering (BBCH 65) delay was 12 days for LWP and 22 days for VLWP, decreasing further at 3 and 13 days at the onset of ripening (BBCH 81). The grape sugar level set for ripening (∼19 °Brix, equivalent to a potential alcohol of ∼11°) was reached by SWP on DOY 238, whereas the same level of ripeness was anticipated by 3 d in LWP and delayed by 6 days in VLWP (**Table 1**). Interestingly, maximum delay over the whole Lorentz et al. (1995) scale occurred at BBCH-79 (i.e., majority of berries touching) with 26 and 36 days for LWP and VLWP as compared to SWP.

**Table 1:** Seasonal evolution (DOY), cumulated degree days (DD) and cumulated net carbon (C) per vine at main phenological stages according to the BBCH coding (Lorentz et al., 1995).

	SWP			LWP			VLWP		
			
BBCH code	DOY^A^	DD^B^ (°C)	C^C^ (g)	DOY	DD (°C)	C (g)	DOY	DD (°C)	C (g)
01 - Beginning of bud swelling	87	7	-5.6	92 (5)	29	-	93 (6)	32	-
03 - End of bud swelling	90	21	-6.9	110 (20)	95	-3.7	126 (36)	204	-6.5
09 - Bud burst	103	59	-11.6	120 (17)	152	-10.4	134 (31)	295	-12.5
15 - 5 leaves unfolded	116	134	-10.7	132 (16)	269	-12.8	143 (27)	369	-16.9
20 - 10 leaves unfolded	133	282	23.4	146 (13)	395	32.1	155 (22)	504	0.44
61 - Beginning of flowering (10% of flowerhoods fallen)	137	323	42.0	149 (12)	423	53.8	160 (23)	588	19.6
65 - Full flowering (50% of flowerhoods fallen)	139	347	53.2	151 (12)	446	68.7	161 (22)	602	22.6
71 - Fruit set	145	387	89.2	154 (9)	486	92.5	167 (22)	678	52.1
73 - Berries groat-sized	151	446	143.1	159 (8)	574	145.7	173 (22)	753	95.7
75 - Berries pea-sized	159	574	225.0	166 (7)	667	212.3	183 (24)	903	182.4
79 - Majority of berries touching	173	753	383.6	199 (26)	1216	601.9	209 (36)	1411	377.9
81 - Beginning of ripening	201	1257	669.6	204 (3)	1320	677.2	214 (13)	1485	413.3
83 - Berries developing color	211	1446	782.2	214 (3)	1485	796.2	218 (7)	1558	448.1
89 - Berries ripe for harvest (19 °Brix)	238	1843	977.3	235 (-3)	1809	1012.4	244 (6)	1842	628.0


*Data refer to cv. Sangiovese grapevines subjected to different timings of winter pruning. SWP, standard winter pruning; LWP, late winter pruning; VLWP, very late winter pruning. ^A^Day of year; ^B^Cumulated DD since DOY 74. ^C^Cumulated carbon (g/vine) since (DOY 74 for SWP) and since DOY 103 and 119, i.e., pruning dates for LWP and VLWP, respectively.*

While the number of days elapsing between bud burst and full bloom (BBCH-09 to BBCH-65) decreased with increasing winter pruning delay (36, 31, and 27 days for SWP, LWP and VLWP, respectively), cumulated heat summation (DD) over the same interval was instead quite similar (288, 294, and 307°C, respectively, **Table 1**). The same number of days (53) was needed by both late pruning levels to transit from full bloom to onset of ripening (BBCH-81) vs. 62 days required by SWP; cumulated DD was still notably close (910, 874, 883 DD for SWP, LWP and VLWP, respectively). The shift from onset of ripening to berries ripe for harvest (BBCH-89) took 37 days in SWP vs. 31 in LWP and 30 days in VLWP, whereas heat requirement for the same transition diminished along with pruning delay (i.e., 586, 489, and 457°C for SWP, LWP and VLWP, respectively). The full season growth cycle between bud burst and ripe berry harvest lasted from a maximum 135 days calculated for SWP to a minimum 113 days for VLWP, with LWP holding an intermediate position (118 days). Notably, while SWP required an ΣDD of 1784°C to complete the path, 1657 and 1647°C were needed by LWP and VLWP, respectively.

**FIGURE 4:**
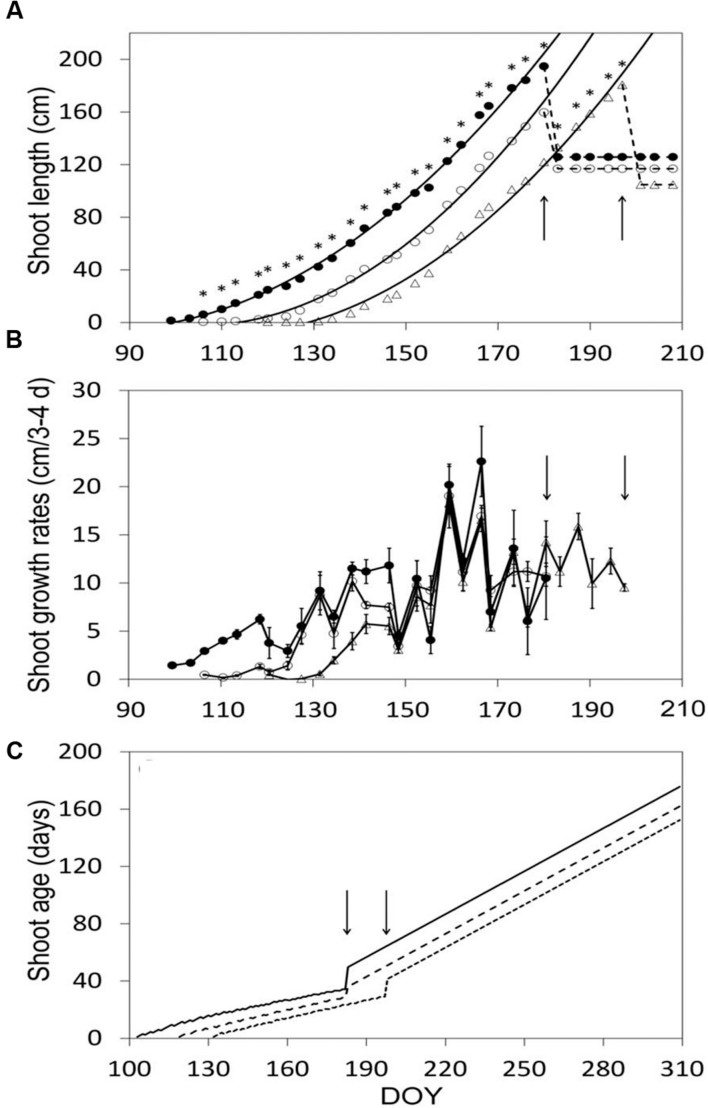
**Shoot elongation (cm, A), shoot growth rates (cm/3–4 days, B) and seasonal mean shoot age (days, C) as affected by winter pruning treatments.** In **(A)** exponential equations fit to shoot elongation vs. DOY data were: *y* = 0.0229x^2^ – 3.8597x + 157.29 (*R*^2^ = 0.996), *y* = 0.0301x^2^ – 6.292x + 326.88 (*R*^2^ = 0.995) and *y* = 0.026x^2^ – 5.6918x + 302.21 (*R*^2^ = 0.992) in SWP, LWP and VLWP treatments, respectively. In **(B)** vertical bars represent standard errors (SE) around means. In all panels, arrows indicate dates of shoot trimming.

After shoot growth had commenced, shoot elongation was at any date different over treatments, and the relative differences stayed almost constant until shoot trimming halted growth (**Figure 4A**). Maximum shoot length pre-trimming was 191, 160, and 181 cm in SWP, LWP and VLWP, respectively (**Figure 4A**). At the respective full bloom (BBCH-65) dates, shoots had ∼12 unfolded leaves, whereas shoot length was 60.4 ± 6.2, 61.1 ± 8.6, and 65.9 ± 6.5 cm in SWP, LWP and VLWP, respectively. Expressing shoot development in terms of growth rates disclosed that upon commencement of shoot growth in VLWP, and despite the favorable thermal regime, pre-bloom growth rates were significantly lower (∼5 cm/3–4 days) than those recorded for the other two pruning dates, ∼10–12 cm/3–4 days (**Figure 4B**). Mean shoot age was consistently modified by treatments, resulting in 34.3 ± 0.3, 28.4 ± 0.9 and 29.1 ± 0.4 at pre-trimming (**Figure 4C**). Trimming caused an expected and abrupt increase in shoot age, estimated at 50.7 ± 0.6, 37.0 ± 2.8, and 41.4 ± 0.6 in SWP, LWP and VLWP, respectively.

### Canopy Function

**FIGURE 5:**
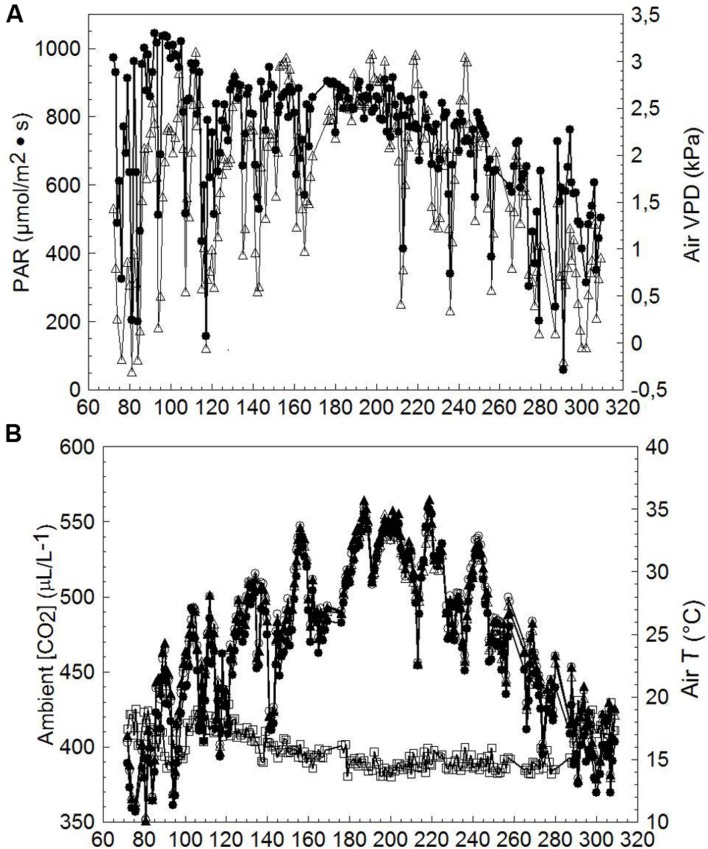
**Seasonal trends of incoming photosynthetically active radiation (PAR, ●), air vapor pressure deficit (VPD, △) (A), inlet chamber air temperature (T, ●), outlet chambers air temperature for SWP (T, ∘), outlet chambers air temperature for LWP (T, ▲), outlet chambers air temperature for VLWP (T, △) and ambient CO_2_ concentration (□) (B) measured at the trial site.** Values are daily means averaged from dawn to dusk.

Mean diurnal PAR and VPD recorded throughout the measuring period (DOY 72-309) notably fluctuated until about DOY 165; thereafter a long series of mostly clear days occurred until treatments were harvested (**Figure 5A**); air VPD peaked occasionally at ∼3 kPa. Average ambient CO_2_ concentration [CO_2_] over the season was 398 ± 12.4 μL/L (mean ± standard deviation), whereas seasonal mean inlet air temperature (T) was 23.8 ± 6.3°C. Seasonal mean T measured at chambers’ outlets was 25.1 ± 5.9°C, 25.1 ± 6.0°C and 24.8 ± 5.7°C for SWP, LWP and VLWP, respectively, corresponding to an overheating contained within 1.3°C for SWP and LWP and 1.0°C for VLWP (**Figure 5B**). An un-seasonably cool period registering inlet mean T of ∼17°C occurred on DOY 141–143.

**FIGURE 6:**
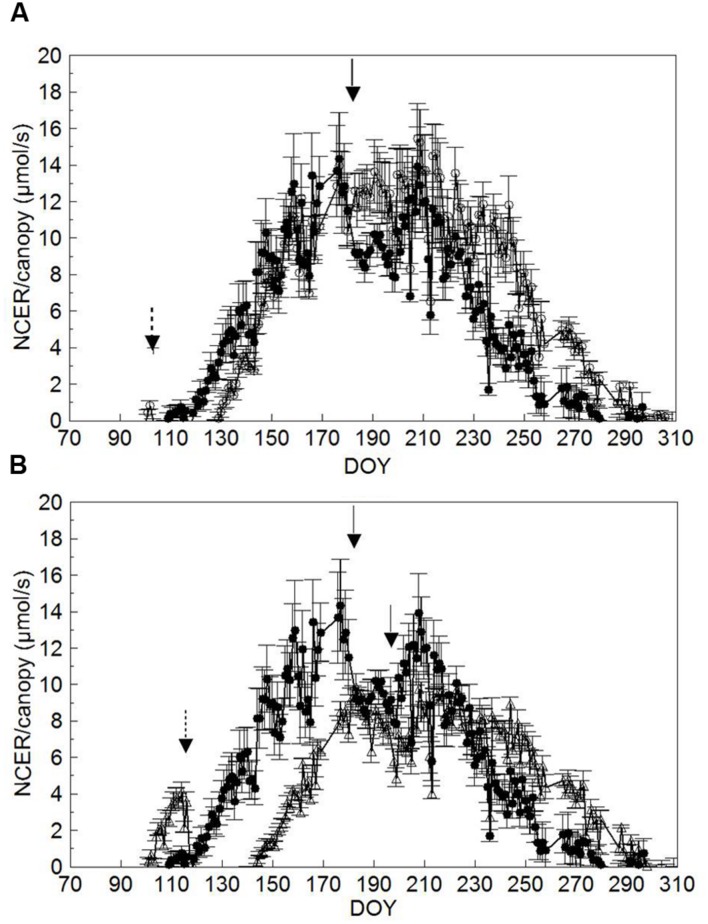
**Seasonal trends of daily mean canopy net CO_2_ exchange rate (NCER, μmol/s) measured in SWP vs. LWP **(A)** and SWP vs. VLWP **(B)** treatments.** Vertical bars indicate standard error (*n* = 4). Dotted arrows indicate dates of LWP and solid arrows dates of shoot trimming.

Comparison of seasonal canopy net CO_2_ exchange rate (NCER) for SWP and LWP shows that SWP started to show a positive NCER on DOY 119 when shoots were around BBCH-16 (**Figures 6A,B**, **Table 1**); the same threshold was reached on DOY 130 and 144 (∼BBCH-15) in both LWP and VLWP. Maximum canopy NCER rates (∼13–14 μmol/s) were recorded in SWP on DOY 176 and 177, i.e., just prior to trimming and close to the BBCH-79 (majority of berries touching). The peak in canopy NCER (∼15.0–15.5 μmol/s) was reached later (i.e., beginning of ripening) in LWP, whereas canopy NCER in VLWP was maximum both before and after shoot trimming with rates close to 9 μmol/s. Shoot trimming performed on DOY 182 in SWP caused a temporary NCER drop of 25%, calculated by comparing mean NCER over 3 days immediately before and after trimming (i.e., 12.2 vs. 9.2 μmol/s). Reduction of canopy NCER in LWP due to trimming was milder (-6%), whereas in VLWP trimming curtailed canopy NCER by 21%. Over the 37-day ripening period (BBCH-81 to BBCH-89) daily mean canopy NCER was 8.8 μmol/s vs. 11.4 μmol/s in SWP and LWP, respectively; VLWP reached a mean NCER of 7.7 μmol/s, 15% less than SWP, over its 33-day ripening period (DOY 214 to DOY 247).

**FIGURE 7:**
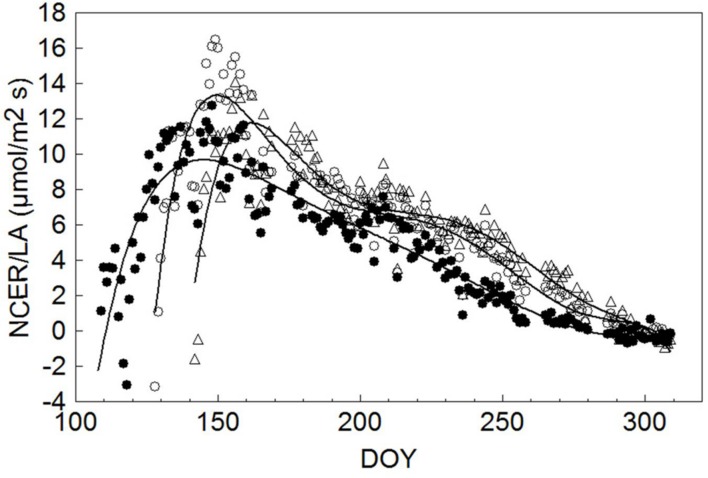
**Seasonal trends of daily mean canopy net CO_2_ exchange rate per unit leaf area (NCER/LA, μmol/m^2^ s) measured in SWP (●), LWP (∘) and VLWP (△).** Each data point is the mean of four vine replicates. For each treatment data were fitted by a six order high precision polynomial curve yielding *R*^2^ = 0.85 in SWP, *R*^2^ = 0.89 in LWP and *R*^2^ = 0.87 in VLWP.

To allow unbiased comparisons, seasonal NCER/LA trends for each treatment were interpolated by a sixth order high precision polynomial curve, indicating that maximum NCER/LA (9.69 μmol/m^2^ s) for SWP occurred on DOY 145, or 34 days after the system started to detect a net CO_2_ gain at DOY 111 (**Figure 7**). Thereafter a steady, yet gradual NCER/LA decline was recorded until net CO_2_ fixation was annulled on DOY 279. In both LWP and VLWP, maximum NCER/LA (13.3 and 11.8 μmol/m^2^s, respectively) was reached 22 days after the commencement of positive CO_2_ fixation and, while a similar gradual decline occurred thereafter, positive CO_2_ differentials were maintained in both pruning dates over DOY 300.

**FIGURE 8:**
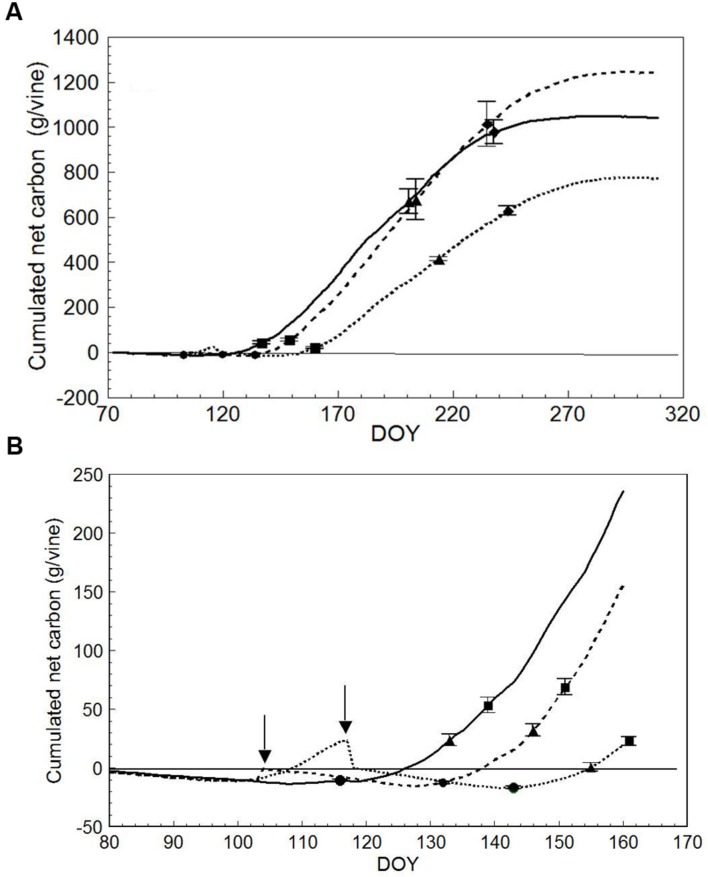
**Seasonal net carbon (C) accumulation **(A)** derived from the canopy photosynthesis monitoring for SWP (solid line), LWP (broken line) and VLWP (dotted line).** On each line, • is for bud burst (BBCH-09), ▲ is for beginning of flowering (BBCH-61), 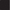 is for beginning of ripening (BBCH-81) and 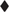 is for berries ripe for harvest (BBCH-89). For each phenological date, vertical bars indicate standard error (*n* = 4). In **(B)**, seasonal net C accumulation is zoomed for early season, • is for five leaves unfolded (BBCH-15), ▲ is for 10 leaves unfolded (BBCH-20) and 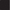 is for full flowering (BBCH**-**65**).** Arrows indicate dates of LWP. Vertical bars indicate SE around means.

Plotting cumulated seasonal net carbon (C) per vine (DOY 72 to DOY 309) resulted in different end-season values of 1243 g/vine in LWP vs. 1042 (-16%) and 772 (-38%) g/vine in SWP and VLWP, respectively (**Figure 8A**). Seasonal dynamics also differed (**Figure 8B**). While LWP pruning performed on DOY 103 slightly improved the net C balance, the opposite was seen in VLWP’s: it had already reached a positive carbon balance the DOY 117 pruning date, i.e., 23.3 g/vine, but abruptly shifted to a negative balance. At BBCH-15, all pruning treatments were still at a negative C balance, whereas at BBCH-20 (10 leaves unfolded) the two earlier pruning treatments were positive (>20 g/vine) and VLWP was still around compensation (0.44 g/vine). These differences were amplified at the onset of flowering, and VLWP showed a much more limited C balance as compared to the other two pruning dates at every phenological stage thereafter. LWP progressively recovered the initial carbon gap when compared to SWP and accumulated carbon values overlapped by DOY 202, i.e., mid-way between veraison and full ripening (BBCH-89). Notably, carbon gain between BBCH 81 and 89 was 308, 358 and 233 g/vine for SWP, LWP and VLWP, respectively. Post-harvest C accumulated until the day of system dismantling, registering 61, 199 and 126 g/vine, respectively.

### Vegetative Growth, Yield and Grape Composition

While shoot number per vine did not differ among treatments, final canopy surface area was lower in VLWP as compared to the earlier pruning dates due to lower primary leaf area and smaller main leaf surface (**Table 2**). Devigoration in VLWP was also confirmed by lower total pruning weight and single cane mass. Total leaf area per vine removed at winter pruning was 3245 ± 202 cm^2^ in LWP and 10945 ± 954 cm^2^ in VLWP, corresponding to 9 and 39% of final total LA/vine, respectively.

**Table 2:** Vegetative growth and pruning weight components recorded on cv. Sangiovese grapevines subjected to different timings of winter pruning.

	Shoots/vine	LA removed at WP(m^2^)	LA removed at ST (m^2^)	Total final LA(m^2^)	Primary LA(m^2^)	Lateral LA(m^2^)	LA of single main leaf(cm^2^)	Cane weight (g)	Total pruning weight (g)	Main canes pruning weight (g)	Lateral pruning weight (g)
SWP	12.3	0c	0.50	3.72a	2.86a	0.86	105.20b	43.2b	528ab	426	102
LWP	10.5	0.32b	0.31	3.64a	2.51ab	1.13	119.65a	62.4a	641a	473	166
VLWP	12.5	1.09a	0.40	2.78b	2.10b	0.68	80.68c	37.5b	455b	390	65
Significance	ns	^∗∗^	ns	^∗^	^∗^	ns	^∗∗^	^∗^	^∗^	ns	ns


*SWP, standard winter pruning; LWP, late winter pruning; VLWP, very late winter pruning. LA, leaf area. ST, shoot trimming. Different lower case letters indicate within column mean separation performed by SNK test at *P* < 0.05 (^∗^) or *P* < 0.01 (^∗∗^). ns indicates non-significance.*

**FIGURE 9:**
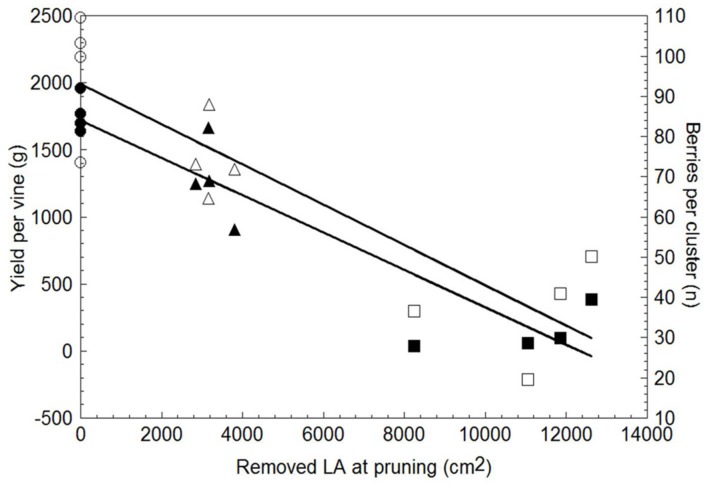
**Relationship between leaf area removed at winter pruning and yield per vine (solid symbols) and total number of berries per cluster (empty symbols).** Within each parameter, data are pooled over treatments (*n* = 12). Linear regression equation for removed LA vs. yield/vine was: *y* = -1397x + 1721.5, *R*^2^ = 0.87; equation for removed LA vs. berry number per cluster was: *y* = -0.005x + 92.995, *R*^2^ = 0.76.

Yield per vine was 28% lower in WLP than in SWP, although at the latest pruning date yield became negligible, i.e., just 145 g/vine (**Table 3**). LWP yield components contributing to the decrease were lower cluster weight and berry number per cluster; VLWP’s main limiting factor was the drop in cluster/shoot to 0.29 and, secondarily, the fact that the few clusters it did develop were also miniaturized as compared to those resulting from the earlier pruning treatments. Although rachis length decreased proportionally with the delay in winter pruning, the concurrent decrease in berries/cluster offset this effect and, regardless of its expression, cluster compacteness showed a decreasing trend the more pruning was delayed, registering significance for VLWP (**Table 3**). Relative variations in vegetative growth and yield components caused the source availability vis-à-vis sink demand to increase the more pruning were delayed (last two columns in **Table 3**). On a more functional basis, a close negative and linear relationship (y = -0.1397x + 1.721.5, R^2^ = 0.87) was found between total LA/vine removed by pruning and yield per vine for data pooled over treatments (**Figure 9**). A less tight, albeit still significant, relationship was also found between removed LA and berry number per cluster at harvest (y = -0.005x + 92.995, R^2^ = 0.76).

**Table 3:** Yield components, cluster compactness and source-to-sink balance indices recorded on cv. Sangiovese grapevines subjected to different timings of winter pruning.

	Yield/vine (g)	Clusters/shoot	Rachis length (cm)	Cluster weight (g)	Cluster number/vine	Total berries/cluster	Berry weight (g)	Seedless berries/cluster	Cluster compactness		Leaf area-to-yield(cm^2^/g)	Yield to pruning weight (g/g)
											
									(g/cm)	(berries/cm)		
SWP	1765a	0.98a	9.94a	154a	12.0a	95a	2.07a	8	12.7a	8.01a	21.1b	3.39a
LWP	1271b	1.31a	7.58ab	98b	13.3a	74b	2.02a	8	11.2a	7.93a	29.7b	2.01b
VLWP	145c	0.29b	6.33b	34c	3.8b	41c	1.35b	11	6.6b	5.68b	379.6a	0.33c
Significance	^∗∗^	^∗∗^	^∗∗^	^∗∗^	^∗∗^	^∗∗^	^∗∗^	ns	^∗∗^	^∗^	^∗∗^	^∗∗^


SWP, standard winter pruning; LWP, late winter pruning; VLWP, very late winter pruning. Different lower case letters indicate within column mean separation performed by SNK test at *P* < 0.05 (*) or *P* < 0.01 (**). ns indicates non-significance.

For the two treatments concurrently picked at first harvest date on DOY 238, SWP fulfilled the expected ripening threshold set at ∼19 °Brix, whereas LWP registered higher concentrations of TSSs, by 1°, total anthocyanins and phenolics; pH and organic acid concentrations were unaffected (**Table 4**). LWP picked 6 d later than SWP (DOY 244), VLWP reached a must sugar concentration at 19.9 °Brix, similar to LWP’s, but total anthocyanins were lower. Interestingly, despite the large source availability as compared to sink demand in VLWP, TA decline was delayed, especially in regard to lower degradation of malic acid.

**Table 4:** Parameters of grape composition recorded at harvest on cv. Sangiovese grapevines subjected to different timings of winter pruning.

	Total soluble solids (°Brix)	pH	Titratable acidity (g/L)	Tartaric acid(g/L)	Malic acid(g/L)	Tartrate-to-Malate ratio	K^+^ (ppm)	Total anthocyanins (mg/g)	Total phenolics (mg/g)
SWP	19.2b	3.45	6.06b	8.20b	1.54b	5.54a	1800	0.43b	2.08b
LWP	20.2a	3.43	5.94b	8.04b	1.60b	5.13a	1759	0.65a	2.70a
VLWP	19.9a	3.45	7.10a	8.95a	2.23a	4.16b	1870	0.48b	2.44a
Significance	^∗∗^	ns	^∗∗^	^∗∗^	^∗∗^	^∗^	ns	^∗∗^	^∗∗^


SWP, standard winter pruning; LWP, late winter pruning; VLWP, very late winter pruning. Different lower case letters indicate within column mean separation performed by SNK test at *P* < 0.05 (*) or *P* < 0.01 (**). ns indicates non-significance.

## Discussion

The main point is understanding if and how retarded winter pruning might induce and maintain consistent ripening delay so as to lead grapes to maturity in a cooler period to offset detrimental effects of global warming (Varela et al., 2015). Since VLWP registered negligible crop, the issue focuses on a comparison of SWP and LWP. That pruning delay elicited an almost corresponding delay in bud burst in our study provides evidence that grapevine is a kind of ideal crop since the apical dominance exerted by the distal nodes on the subtending ones is very strong and stable (**Figure 2**). Indeed, it explains why the 16 days time/lapse between the two late pruning dates was very close to their 14 days time difference in bud burst (**Table 1**). Note, too, that the initial, consistent bud-break delay registered in LWP (+ 17 days vs. SWP) was fully offset at ripening.

Looking at chronological and thermal lapses between development stages shows that most of the recovery by LWP and VLWP took place from bud burst to full bloom and from the latter to the onset of ripening (**Table 1**). Since heat summation required to complete these shifts was very similar over treatments, it is apparent that the late-burst treatments benefited from higher post-bud burst temperatures, thereby allowing them to reach the required DD threshold in fewer days. These data confirm air temperature as the main driver and better predictor of bloom date than bud burst.

In a comprehensive study carried out over 7 years on 114 cultivars, McIntyre et al. (1982) evaluated the consistency of various climate indicators as time predictors of specific phenological stages in the bud burst-to-bloom window and found that cumulative maximum temperature had the lowest coefficient of variation (3.8) and number of days had the highest (8.4). More recently, a comparison of models for studying grapevine phenology under present and future climate scenarios reported that better calibration was found for the early stages that are usually more temperature-driven (Fila et al., 2012). Interestingly, despite marked variation in full-bloom date between our treatments, the number of unfolded leaves counted on shoots at BBCH 65 was the same (12), confirming, as reported by Coombe (1980), that flowering in grapes truly occurs at a given number of formed internodes regardless of shoot length at that specific time.

Temporal sequence of phenological stages as affected by our treatments has two major practical implications. First, that pruning at BBCH 12 (LWP) resulted in a long bud-burst delay (17 days), which seems effective if the goal is to diminish the risk of incurring spring frost; conversely, if the target is also to postpone full ripening, LWP seems unlikely to be effective since the Winkler index of 2291°C in our trial environment offers a great deal of compensation throughout the remainder of the season.

Pruning date also notably altered seasonal photosynthetic dynamics and canopy efficiency (**Figures 6** and **7**). It is well known that upon bud burst the early stages of vine shoot growth are entirely fueled by starch mobilization from the perennial organs (Lebon et al., 2008). Intensity of mobilization progressively fades until the onset of net photosynthesis in the canopy (Zapata et al., 2004). Looking at NCER/canopy trends, we see that the onset of net NCER, i.e., the transition from a sink to source function, took place at the stage of 5–6 unfolded leaves on the shoot across all treatments (**Figure 6**). This information seems a quite useful update of previous reports indicating that a single leaf becomes a source organ when it reaches between one-third and one-half of final size (Koblet, 1969; Petrie et al., 2000).

Since leaf size varies depending upon node position on the stem, scaling up such information to shoot or canopy leaves is very cumbersome. Generally speaking, our findings for net canopy CO_2_ thresholds are in agreement with data from Lebon et al. (2004, 2005) and Zapata et al. (2004) indicating that the commencement of net CO_2_ is synchronous with female meiosis in Pinot Noir, which takes place at BBCH-15 + 2–8 days. Conversely, our results do not confirm that maximum NCER occurs at bloom at canopy scale, as stated in Lebon et al. (2008). Maximum NCER in all our treatments was reached before trimming (i.e., pea size stage) and post-trimming levels were similarly high until the onset of berry color, a window of some 50 days. It is quite clear that, regardless of differences in shoot age, the balance between younger, fully expanded apical leaves and basal ones still retaining good rates of photosynthesis is optimal and, hence, conducive to maximum canopy CO_2_ assimilation.

Expressing canopy NCER per-unit of leaf area (LA) is a true measure of photosynthetic efficiency of the leaf tissue since confounding effects due to variations in vine size, light exposure, age, degree of healthiness typical of single-leaf sampling are avoided. The trends in **Figure 7** show that it took 34 days for NCER/LA in SWP to peak at around 10 μmol m^-2^s^-1^. This age strongly supports previous work based on single-leaf assessment showing that maximum leaf P_n_ rates are reached at around 35–40 days of age, a window slightly in excess of the time a leaf needs to complete expansion (Kriedemann, 1968; Poni et al., 1994). Interestingly, our data confirm that a progressively gradual decline sets in once the peak is reached. This leaf longevity pattern seems rather peculiar to sites affected by high summer temperature. In fact, peak leaf photosynthesis is maintained for longer periods of time when evaluated in cooler climates as was found for Chasselas and Riesling grown in northern European sites (Schultz et al., 1996; Zufferey and Murisier, 2002).

More importantly, both LWP dates considerably reduced the time the foliage needed to reach maximum efficiency (22 days). Anticipating the peak in canopy assimilation per leaf area unit is a totally new and interesting finding and pertains to the mechanisms that the grapevine uses to adapt to unusually late pruning. One possible explanation for this response is that the higher heat summation available during leaf growth for the two late prunings shortened the time leaves needed to reach full size and, hence, maximum photosynthetic potential. As a matter of fact, DD accumulated over bud-break date + 30 days were 231, 288, and 362°C for SWP, LWP and VLWP, respectively. Noteworthy too is that both LWP and VLWP had NCER/LA rates higher than SWP’s at the peak. It is quite well known that leaves developing under higher temperatures can develop a more efficient photosynthetic apparatus primarily because the transpiration stream is more efficient at supplying water, hormones and solutes to the aerial part (Flore and Lakso, 1988). Lastly, when the gradual post-peak decline set in, NCER/LA rates on LWP and VLWP were higher until the end of season so that the late pruning levels could benefit from an extra month of positive net CO_2_ uptake as compared to SWP, whose last positive NCER/LA value was recorded on DOY 279. Taken together, these three compensation mechanisms, i.e., faster attainment and higher seasonal NCER/LA peak and slower late season senescence, all contributed to photosynthetic recovery capacity in late-pruned treatments and, ultimately, explain why even an ample initial delay in growth commencement is difficult to maintain.

The most significant impact that LWP had on vine response regarded yield and its components. Although the impact of the technique on yield will have to be fortified by longer term field studies where the role played by other factors (i.e., the size of the reserve storage pool and the effects that an early source limitation might have on next season bud induction), in our conditions, delaying pruning until BBCH-17 almost offset yield by drastically reducing both cluster number per vine and berry number per cluster. Since it is conceivable that the number of cluster primordia contained in the dormant buds was similar over treatments, attention should focus on those factors inhibiting the conversion of inflorescence primordia to complete inflorescences. According to Carmo Vasconcelos et al. (2009), this process resumes as shoot development begins in the spring during the second season of the bud differentiation cycle and, according to Bernard and Chaliès (1987), continues for 15–20 days after bud burst in cvs. Grenache and Carignan. If the BBCH 09 + 20 days period is considered, it is evident that the compensation point for CO_2_ exchange was reached at the very end of this window (**Figure 8B**). Thus the severe source limitation caused by VLWP may have caused pre-developed inflorescence primordia to revert to tendrils. Previous studies have reported that this phenomenon is indeed possible and phosphorous supply (Skinner and Matthews, 1989), early water deficit (Matthews and Anderson, 1989) and application of exogenous gibberellin (Yahyaoui et al., 1998) are among the causal factors involved. Visual monitoring of developing shoots in VLWP rules out the possibility that the phenomenon of filage (verrankung), flower abortion before anthesis with the reversion of the inflorescence to a tendril, took place (Champagnol, 1984).

In LWP, a 26% reduction in yield per vine was registered, yet due to a different regulation mechanism. In fact, since clusters/shoot, cluster number per vine and berry weight did not differ as compared to SWP, total berry number per cluster only was responsible for the yield constraint (**Table 3**). Fewer berries per cluster at harvest can derive from multiple factors involving both determinism of initial flower number per cluster and fruit-set, which in turn is influenced by a series of factors (May, 2004). Although flower number per cluster was not determined in our study, it is very unlikely that fruit set had been differentially affected by treatment since no adverse weather or severe source limitation was recorded either before or after flowering. Dunn and Martin (2000) in a study on Cabernet Sauvignon vines used delayed pruning to induce bud-burst under different temperature conditions and found that flower number per inflorescence decreased slightly when soil and air temperatures were higher for the days around bud-burst. This did not seem to hold for the SWP vs. LWP comparison of our study since mean air T calculated over three days around bud-burst resulted in 17.3 and 14.3°C, respectively.

Rather, vine hormonal status seems to be at work. It is well established that buds made terminal by pruning produce more flowers per cluster than buds in similar node positions but which are not terminal (May, 2004). It is likely that flower formation of the proximal shoots is directly inhibited by auxin imported from the distal shoots which grew earlier (May, 2004). On the other hand, it is also well known that during the second season of the bud differentiation cycle, gibberellins (GAs) cause floral inhibition in Vitis vinifera (Srinivasan and Mullins, 1981). Reproductive growth in the shoot is not totally inhibited by GAs because uncommitted primordia are still made, indicating that the GA signaling acts later by inhibiting the production of floral meristems (Boss and Thomas, 2002). Moreover, GAs are also known to enhance flower drop in grapevine (Domingos et al., 2015; Giacomelli et al., 2015). Overall, endogenous GSs produced during the initial flush of growth in the LWP treatment could be a likely candidate to explain the reduction in berry number per cluster recorded at harvest.

## Conclusion

Hypothesis that late or VLWP applied after commencement of spring growth can consistently delay the whole annual growing cycle in the grapevine was partially confirmed. Delay was very pronounced at bud-burst (+31 days in VLWP vs. C) and significant until fruit-set; hereafter under the warm conditions of our trial site significant compensation occurred. Compensation was especially strong in the LWP treatment due to a shorter time needed to reach maximum NCER/leaf area, highest maximum NCER/leaf area (+37% as compared to SWP) and higher NCER/leaf area rates from veraison to end of season. As a result, seasonal cumulated carbon in LWP was 17% higher than SWP. A negative linear relationship found between total leaf area per vine removed by LWP and yield per vine preliminarily suggests that no more than 10% of the final leaf area should be removed to prevent excessive yield reduction, thereby making the technique economically unsustainable. Albeit such relationship will have to be strengthened in longer term field studies which, besides including year-to-year variation, can accommodate effects due to different size of storage pools as well as carry-over effects on next season bud induction and differentiation, our LWP response suggests that this treatment may prove to be an excellent substitute for the costly and not entirely reliable cluster thinning practice.

## Author Contributions

MG, SP designed and supervised the research; MG, FP, GC, MM performed the research and analyzed the data; MG, SP drafted the manuscript; FP, GC, MM contributed to the whole canopy gas exchange assessment; MG, SP, AP, ST critically revised the manuscript. All authors read and approved the final manuscript.

## Conflict of Interest Statement

The authors declare that the research was conducted in the absence of any commercial or financial relationships that could be construed as a potential conflict of interest.
